# E-health literacy and health system distrust: exploring a nonlinear association in a cross-sectional study

**DOI:** 10.1590/1806-9282.20252015

**Published:** 2026-08-21

**Authors:** Ogulcan Come, İrem Yaren Bas, Vildan Mevsim

**Affiliations:** 1 Dokuz Eylul University – İzmir, Türkiye.

**Keywords:** Health literacy, Trust, Digital health, Regression analysis

## Abstract

**BACKGROUND:**

E-health literacy is essential for navigating digital health resources and making informed health decisions. Although higher literacy is assumed to foster institutional trust, its relationship with health system distrust remains unclear.

**OBJECTIVE:**

The aim of this study was to examine the association between e-health literacy and health system distrust, considering possible nonlinear patterns and sociodemographic moderators.

**METHODS:**

A cross-sectional survey was conducted among 384 adults in our institution using the e-Health Literacy Scale and the Health System Distrust Scale. Sociodemographic data and digital device use were recorded. Nonparametric comparisons, multiple linear, and quadratic regression analyses were performed, adjusting for confounders.

**RESULTS:**

E-health literacy differed significantly by education, socioeconomic status, employment, and device use (p<0.05). Distrust varied by socioeconomic and marital status. Linear regression showed no significant association between e-health literacy and distrust (p=0.552). After adding a quadratic term, a statistically significant nonlinear association emerged (p<0.001), with predicted distrust varying across levels of e-Health literacy. High socioeconomic status independently predicted lower distrust (B=-4.97, p=0.002).

**CONCLUSION:**

E-Health literacy shows a modest, nonlinear association with health system distrust in this sample. However, the small explained variance indicates that distrust is largely shaped by factors beyond digital health competence. Further research is needed to clarify underlying mechanisms.

## INTRODUCTION

The digitalization of healthcare has transformed how people access and use health information. The rise of telemedicine, electronic health records, and mobile health tools has increased individuals’ responsibility for managing their own health^1^. Consequently, e-health literacy—the ability to find, understand, assess, and apply digital health information—has become crucial^2^. It shapes informed decision-making, health behaviors, communication with professionals, and navigation within healthcare systems^3^.

Growing evidence indicates that individuals with higher e-health literacy are more likely to engage in preventive health behaviors, manage chronic conditions effectively, and utilize health services appropriately^4^. Such individuals tend to cross-check digital content with trusted medical sources, participate more actively in medical consultations, and avoid misleading or inaccurate health information^5^. Conversely, individuals with limited e-health literacy may face challenges in discerning credible content online, struggle with interpreting digital health information, and may be disproportionately affected by misinformation^6^.

Individuals with low levels of e-Health literacy often rely primarily on direct communication with healthcare professionals, family physicians, nurses, or official health institutions for health information rather than on independent searches of digital sources. They are more likely to obtain information through face-to-face consultations, printed materials, or announcements from authorized organizations^7^. Low e-Health literacy is more common among older adults, individuals with lower educational attainment, lower socioeconomic status, limited digital access, and those living in rural or underserved areas^8^. As e-Health literacy increases, individuals are more likely to actively seek health information online, but may not yet possess sufficient critical appraisal skills to consistently distinguish evidence-based content from misinformation, conspiracy narratives, or false health beliefs^9^. Exposure to narratives such as vaccine hesitancy or refusal, exaggerated claims about hidden harms of medical treatments, or distrust-oriented conspiracy theories targeting health authorities may increase skepticism toward healthcare institutions^10^. At higher levels of e-Health literacy, however, individuals become more capable of evaluating source credibility, cross-checking information with scientific and official sources, and resisting misinformation^9^.

Beyond individual-level behaviors, the experience of engaging with digital health systems may also influence broader attitudes toward healthcare institutions. In this context, health system trust—defined as an individual's confidence in the competence, honesty, and fairness of the healthcare system—has emerged as a key psychological construct shaping healthcare utilization and adherence^3^. Trust in the healthcare system has been linked to greater patient satisfaction, improved adherence to treatment, and enhanced willingness to seek preventive care^11,12^. In contrast, health system distrust has been associated with delays in care, reduced uptake of health interventions, avoidance of medical settings, and lower participation in public health programs^12^.

As digital health technologies become more integrated into clinical practice and everyday health management, understanding the potential interplay between e-health literacy and perceptions of the health system is of growing importance.

Despite its conceptual relevance, the empirical relationship between e-health literacy and health system trust/distrust has not been extensively examined. In light of the increasing digitization of healthcare services and the growing concerns around public confidence in health systems—particularly following global health crises such as the COVID-19 pandemic—this gap warrants further investigation.

Against this backdrop, the present study aims to examine the relationship between e-health literacy and health system distrust among adults. By investigating whether different levels of digital health competence are associated with varying degrees of trust in the healthcare system, the study seeks to inform the design of more effective health communication strategies and literacy-based interventions. These insights may ultimately contribute to fostering a digitally empowered yet trustworthy relationship between the public and the evolving health system.

## METHODS

### Study design and setting

This study employed a cross-sectional analytical design and was conducted entirely online. Data were collected using a structured questionnaire created in Google Forms and disseminated via digital communication channels and social media platforms (e.g., Instagram, WhatsApp, X/Twitter, Facebook, and email). No face-to-face data collection was carried out.

### Participants and sampling

The study population consisted of individuals aged 18 years and older. A non-probability snowball sampling method was used for participant recruitment. Data collection was conducted online in July–August 2025 using a structured questionnaire disseminated via Google Forms. A non-probability snowball sampling method was used for recruitment. Because this sampling approach does not support population prevalence estimation, the target sample size was guided by regression modeling considerations rather than margin-of-error calculations. A power calculation for multiple regression (α=0.05, power=0.80) indicated that approximately 173 participants would be sufficient to detect a small-to-moderate effect size (f^2^=0.05) with 15 predictors. To ensure stable estimation of regression coefficients, particularly for the quadratic term, and to increase analytical precision, recruitment continued until 384 complete responses were obtained.

Inclusion criteria were (1) being 18 years or older, (2) consenting to participate voluntarily, (3) completing the questionnaire fully and consistently, and (4) having internet access to complete the online survey. The exclusion criterion was submission of incomplete or invalid responses, specifically cases in which only one answer option was selected throughout the entire questionnaire. Based on this criterion, five participants were excluded from the analysis.

### Data collection tools

The online questionnaire consisted of three sections:

Sociodemographic Information Form:

Collected data on age, gender, education level, marital status, employment status, occupation, socioeconomic status, and frequency of digital device use.

Health System Distrust Scale (HSDS):

Originally developed by Rose et al., the HSDS is a 10-item instrument designed to assess individuals’ distrust toward healthcare institutions and systems^13^. The scale measures perceptions related to confidentiality, unethical experimentation, lack of transparency, and prioritization of institutional interests over patient needs. Items are rated on a 5-point Likert scale. In the Turkish validation study, the scale demonstrated a unidimensional structure and satisfactory internal consistency (Cronbach's alpha=0.789)^14^. Items 2, 8, and 9 are reverse coded. Higher total scores indicate greater distrust toward the healthcare system.

e-Health Literacy Scale (eHEALS):

Developed by Norman and Skinner and adapted into Turkish by Uskun et al., this 8-item scale measures individuals’ perceived skills in finding, evaluating, and using online health information^15,16^. Items are rated on a 5-point Likert scale. Higher total scores reflect greater perceived e-health literacy. The Turkish version of the scale has shown good internal consistency (Cronbach's alpha=0.88)^16^.

### Variables

Independent variables in this study were participants’ e-health literacy level, measured using the total score of the eHeals. Additional independent variables included sociodemographic characteristics such as age, gender, education level, marital status, employment status, and frequency of digital device use. The dependent variable was the level of health system distrust, assessed using the total score of the HSDS.

### Ethical considerations

Ethical approval for the study was obtained from our university ethics committee, Document No: 9866-GOA on 09.07.2025 with decision number 2025/23-07. Participants were informed about the study's purpose, and electronic informed consent was obtained prior to participation. All responses were collected anonymously, and data confidentiality was strictly maintained.

### Statistical analysis

Descriptive statistics were used to summarize participants’ sociodemographic characteristics and scale scores. The normality of continuous variables was evaluated using skewness–kurtosis values and the Shapiro-Wilk test. Depending on the distribution, either parametric or nonparametric tests were used.

To assess the relationship between e-health literacy and health system distrust scores, both linear and quadratic regression analyses were performed to identify potential nonlinear associations. The quadratic model was used to evaluate whether different levels of e-health literacy were associated with distinct patterns of distrust compared to low or high literacy levels.

Comparisons of eHEALS and HSDS scores across sociodemographic subgroups were conducted using the Mann-Whitney U test for two-group comparisons and the Kruskal-Wallis H test for comparisons among three or more groups. All analyses were performed using SPSS version 26.0 (IBM Corp., Armonk, NY, USA). A two-tailed p<0.05 was considered statistically significant.

## RESULTS

A total of 384 valid responses were analyzed. Descriptive comparisons of e-Health literacy and health system distrust scores across sociodemographic groups are presented in Table 1. Overall, e-Health literacy scores differed significantly by education level, marital status, employment status, socioeconomic status, and digital device usage (p<0.05 for all), while health system distrust scores varied significantly by marital status and socioeconomic status (p=0.020 and p=0.002, respectively). Participants with higher education and socioeconomic status reported significantly greater e-Health literacy and lower levels of distrust. Single individuals demonstrated significantly lower health system trust compared to married participants. Employment status was associated with e-Health literacy, with employed individuals showing higher scores. Device usage frequency was also associated with e-Health literacy (p=0.041), with regular users reporting higher scores than those who used devices occasionally or not at all. In contrast, no significant differences were observed in e-Health literacy or distrust scores based on gender or chronic disease status.

**Table 1:** Comparison of e-Health score and Health System Distrust Scale across sociodemographic variables.

Variable	Category	e-Health literacy [median (Min–Max)]	p-value	Distrust score [median (Min–Max)]	p-value
Gender	Male	30 (8–40)	0.990	30 (15–46)	0.275
	Female	30 (8–40)		28 (15–44)	
Education	Literate*	15.5 (15–16)	<0.001	25 (24–26)	0.310
	Primary school	25 (8–40)		29 (20–40)	
	High school	26.5 (8–40)		29 (17–46)	
	University and above	31 (8–40)		29 (15–46)	
Marital status	Single	32 (8–40)	<0.001	28 (15–44)	0.020
	Married	29 (8–40)		30 (15–46)	
Employment	Employed	30 (8–40)	0.017	29 (15–46)	0.266
	Unemployed	24 (8–37)		29 (17–43)	
	Retired	30 (8–40)		29.5 (16–46)	
	Student	29 (8–40)		27 (15–37)	
Socioeconomic status	Low	27 (8–40)	0.025	34 (16–43)	0.002
	Middle	30 (8–40)		30 (15–46)	
	High	32 (8–40)		27 (15–42)	
Chronic disease	Yes	30 (8–40)	0.909	31 (15–46)	0.063
	No	30 (8–40)		28.5 (15–46)	
Device use	Not using	16 (8–32)	0.041	24 (21–27)	0.208
	Occasionally	27 (8–40)		27 (20–40)	
	Regularly	30 (8–40)		29 (15–46)	

* individuals who can read and write but have not completed any formal level of schooling.

A multiple linear regression was conducted to examine whether e-Health literacy and various sociodemographic variables significantly predicted health system distrust scores. The model, which included gender, education level, marital status, employment status, socioeconomic status, chronic disease status, digital device use, and e-Health literacy score, was statistically significant, F(14, 370)=2.238, p=0.006. The overall model accounted for approximately 7.8% of the variance in distrust scores (R^2^=0.078, adjusted R^2^=0.043) (Table 2).

**Table 2:** Multiple linear regression predicting Health System Distrust Scale.

Predictor	B	SE	t	p-value	95%CI for B
(Intercept)	22.19	5.97	3.72	<0.001	(10.46, 33.93)
Gender (female)	-1.17	0.68	-1.71	0.088	(-2.51, 0.18)
Education (primary school)	5.29	4.83	1.10	0.273	(-4.20, 14.78)
Education (high school)	5.85	4.69	1.25	0.214	(-3.38, 15.08)
Education (university or higher)	4.93	4.74	1.04	0.299	(-4.39, 14.25)
Marital status (married)	0.96	0.73	1.31	0.190	(-0.48, 2.40)
Employment (unemployed)	0.32	1.44	0.22	0.826	(-2.51, 3.14)
Employment (retired)	0.70	0.90	0.78	0.438	(-1.07, 2.46)
Employment (student)	-1.48	1.45	-1.03	0.306	(-3.42, 1.36)
Socioeconomic status (middle)	-2.85	1.52	-1.88	0.061	(-5.84, 0.14)
Socioeconomic status (high)	-4.97	1.62	-3.06	0.002	(-8.16, −1.77)
Chronic disease (yes)	0.73	0.74	0.99	0.321	(-0.72, 2.18)
Device use (occasionally)	4.90	3.78	1.29	0.196	(-2.54, 12.33)
Device use (regularly)	6.19	3.67	1.69	0.093	(-1.03, 13.42)
e-Health literacy score	-0.02	0.04	-0.60	0.552	(-0.09, 0.05)

Note: Reference categories: Gender: male; Education: Illiterate; Marital status: single; Employment: employed; Socioeconomic: low; Device use: not using. SE: standard error; CI: confidence interval.

Among the predictors, high socioeconomic status was significantly associated with lower distrust scores (β=-4.97, p=0.002, 95%CI −8.16, −1.77). Other predictors—including e-Health literacy (p=0.552), gender (p=0.088), and digital device usage—were not statistically significant in the model.

To address concerns regarding multicollinearity between the linear and quadratic terms, e-Health literacy scores were mean-centered before creating the squared term. Variance inflation factors (VIFs) were examined and indicated low multicollinearity (VIF=1.47 for both predictors).

The quadratic regression model predicting health system trust was statistically significant, F(2, 382)=9.93, p<0.001, explaining approximately 4.9% of the variance in trust scores (R^2^=0.049; adjusted R^2^=0.044). A nested model comparison showed that adding the quadratic term significantly improved model fit compared to the linear model [ΔF(1, 382)=18.17, p<0.001], indicating that the quadratic model provides a significantly better fit than the linear model.

**Figure 1:**
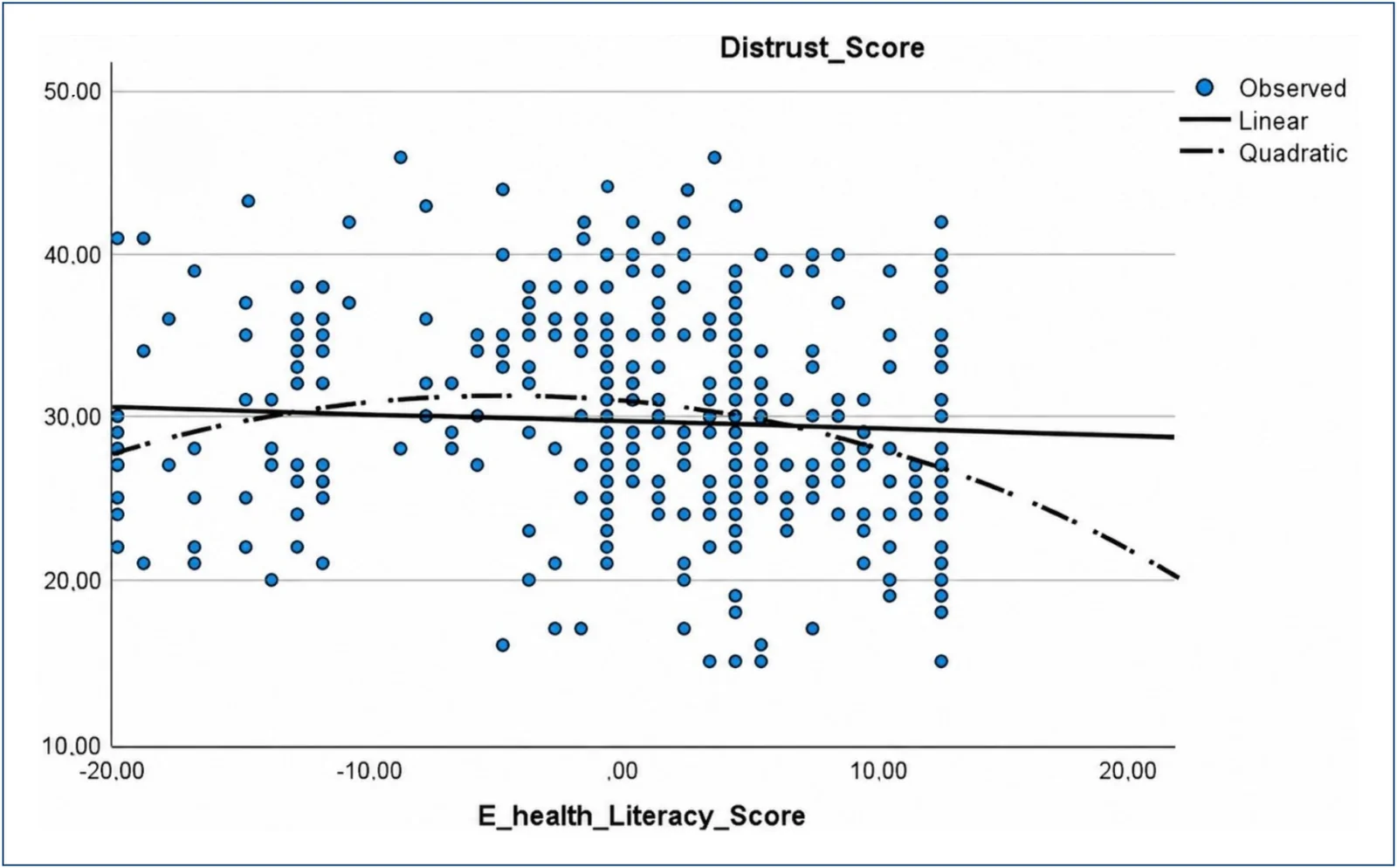
Relationship between mean-centered e-Health literacy and health system distrust. Dots represent observed individual scores. The solid line shows the fitted linear regression model, and the dashed line shows the fitted quadratic regression model. The quadratic curve illustrates the nonlinear association between e-Health literacy and distrust, with distrust peaking around a centered value of −4.66 (corresponding to an original e-Health literacy score of approximately 23.2).

Both the linear and quadratic terms of mean-centered e-Health literacy were statistically significant. The linear term was negatively associated with trust (B=-0.149, SE=0.043, β=-0.211, p<0.001), and the quadratic term was also negative (B=-0.016, SE=0.004, β=-0.258, p<0.001), indicating a nonlinear association characterized by an overall decreasing pattern with increasing curvature (Table 3). The vertex of the curve was calculated as -b_1_/(2b_2_), yielding a centered value of −4.66, corresponding to an original e-Health literacy score of approximately 23.2. This represents the point at which predicted distrust reaches its maximum. This suggests that higher levels of e-Health literacy are associated with lower levels of health system distrust and that this decline becomes steeper at higher literacy levels (Figure 1).

**Table 3:** Quadratic regression analysis predicting health system distrust.

Predictor	B	SE	B	t	p	95%CI for B	VIF
Constant	30.898	0.431	–	71.659	<0.001	(30.050, 31.746)	–
e-Health literacy (centered)	-0.149	0.043	-0.211	-3.482	<0.001	(-0.234, −0.065)	1.47
e-Health literacy (squared) (centered)	-0.016	0.004	-0.258	-4.263	<0.001	(-0.023, −0.008)	1.47

Note: Dependent variable: Health System Distrust Score. e-Health literacy scores were mean-centered prior to creating the squared term. All VIF values were below 2, indicating low multicollinearity. SE: standard error; CI: confidence interval; VIF: variance inflation factor.

## DISCUSSION

This study explored the association between e-health literacy and health system distrust using linear and quadratic models. After correcting for multicollinearity through mean-centering, both the linear and quadratic terms remained significant, indicating that the relationship is not strictly linear. However, the direction of the coefficients suggests an overall decreasing pattern, meaning that higher levels of e-Health literacy are associated with lower levels of distrust, with the decline becoming steeper at higher literacy levels^17,18^.

These results align with evidence that health literacy's effects are not uniformly protective and depend on cognitive and contextual factors^5,19^. Possessing basic digital skills but limited evaluative ability may misinterpret online information and show greater distrust; highly literate users employ source verification and critical navigation, forming more balanced judgments^5,20^.

Sociodemographic trends confirmed digital inequities: higher education, socioeconomic status, and regular device use correlated with greater e-health literacy, reflecting structural disparities in access and opportunity^21,22^. Distrust scores varied only by socioeconomic and marital status, with higher-income and married participants showing less distrust—likely due to social capital and better healthcare access^23,24^.

Although education predicted literacy, it did not predict distrust, implying that formal schooling enhances digital competence more than institutional trust, which may depend on personal or sociopolitical experiences^5^.

Also, the results should be interpreted within the Turkish healthcare context, which offers broad population coverage, strong primary care, and widely used digital platforms such as e-Nabız and MHRS^14,16^. These features support access, continuity, and transparency, which may foster baseline trust, especially among individuals relying on official institutions and healthcare professionals. Highly e-Health-literate individuals may benefit most from these systems by effectively using official digital services. Because healthcare structures and digital infrastructures differ across countries, the findings may not directly generalize to other settings^9^.

### Limitations

This study has several limitations. This study used non-probability snowball sampling, which is prone to selection bias and does not produce a population-representative sample. Although the sample size was sufficient for regression analysis, it does not support population-level inference. Online data collection may have underrepresented individuals with very low digital access or skills. Finally, the cross-sectional design prevents causal interpretation of the observed associations. Its cross-sectional design prevents causal inference between e-health literacy and trust. Self-reported data may involve bias, and findings from a single national context may limit generalizability to other healthcare systems.

The explained variance of the models was modest (adjusted R^2^≈0.044 for the quadratic model), indicating that most variability in health system distrust is driven by factors not measured here. Therefore, e-Health literacy should be viewed as one contributing factor among many, with limited standalone explanatory power.

## CONCLUSION

This study identifies a statistically significant but modest nonlinear association between e-Health literacy and health system distrust. While the quadratic model suggests that distrust varies across levels of digital health competence, the small proportion of explained variance indicates that e-Health literacy accounts for only a limited share of distrust.

### Future studies

Future studies should examine a broader range of factors influencing health system distrust, including healthcare experiences, institutional transparency, media exposure, and sociopolitical context. Mixed-methods, subgroup, and longitudinal designs are needed to clarify how e-Health literacy interacts with these factors and whether nonlinear patterns are robust.

## DECLARATION OF GENERATIVE AI AND AI-ASSISTED TECHNOLOGIES

During the preparation of this work, the author(s) used ChatGPT-4o (OpenAI, 2025) in order to improve grammar, language clarity, and phrasing. After using this tool/service, the author(s) reviewed and edited the content as needed and take(s) full responsibility for the content of the publication.

## Data Availability

The datasets generated and/or analyzed during the current study are available from the corresponding author upon reasonable request.
